# Evaluating the Potential of Reasoning Large Language Models to Perpetuate Racial and Gender Disease Stereotypes in Health Care

**DOI:** 10.2196/82256

**Published:** 2026-05-28

**Authors:** Joshua J Docking, Lee X Li, Bradley D Menz, Stephen Bacchi, Ashley M Hopkins, Michael J Sorich

**Affiliations:** 1 College of Medicine and Public Health, Flinders University, GPO Box 2100, Adelaide, SA, 5001, Australia, 61 (08) 82013217; 2 Adelaide Medical School, Adelaide University, Adelaide, SA, Australia

**Keywords:** large language model, reasoning LLM, artificial intelligence, bias, stereotypes, fairness, health equity, race, gender, representation

## Abstract

This evaluation of 36,000 clinical vignettes found that next-generation reasoning large language models, o3-mini and DeepSeek-R1, frequently perpetuate racial and gender stereotypes for common medical conditions, indicating that advancements in reasoning do not inherently improve representational fairness.

## Introduction

Large language models (LLMs) have the potential to transform health care but risk exacerbating health disparities if they perpetuate biases [1-3]. Zack and colleagues [4] demonstrated potential racial and gender biases in clinical vignettes generated by GPT-4, including overrepresentation of Black patients in stereotypical medical conditions. Since then, next-generation reasoning LLMs have emerged, offering improved reasoning capability (“thinking” before answering), with this model class demonstrating superior benchmark performance [5]. Whether this will reduce representational bias in health care remains unknown. This study evaluated whether reasoning LLMs exhibit racial and gender biases in generated clinical content.

## Methods

Using the methods of Zack et al [4], two prominent reasoning LLMs of distinct geographic origin, o3-mini (OpenAI) and DeepSeek-R1 (DeepSeek; 671B full model), generated patient cases across 18 medical conditions that represent a spectrum of demographic-prevalence relationships (Multimedia Appendix 1), specifying a US population, using 10 prompt variations (Table S1 in Multimedia Appendix 1), and running 100 times each. Patient demographic characteristics in the generated vignettes were extracted using the methods of Zack et al [4], and the proportion of race and gender representation for each condition was calculated. A qualitative analysis of DeepSeek-R1’s reasoning traces was also performed on a random sample of 20 vignettes (Multimedia Appendix 1). Misrepresentation (LLM estimate minus the published US epidemiological estimates [4]) was summarized as the median (range) across 18 medical conditions. For example, if 60% of LLM-generated HIV cases were Black patients, compared to the 40% of Black patients reported in representative US studies of HIV (Table S2, Multimedia Appendix 1), misrepresentation would be +20%. Positive values indicate overrepresentation, and negative values indicate underrepresentation. A difference greater than 20% was considered the threshold for significant misrepresentation, indicating a practically meaningful deviation in demographic representation. Sensitivity analyses using 10% and 30% thresholds confirmed that findings were robust to threshold selection (Multimedia Appendix 1). *χ*^2^ goodness-of-fit tests with Benjamini-Hochberg false-discovery rate correction were used to assess whether the LLM-generated demographic distributions differed significantly from epidemiological baselines for each condition (Multimedia Appendix 1).

## Results

**Figure 1.**
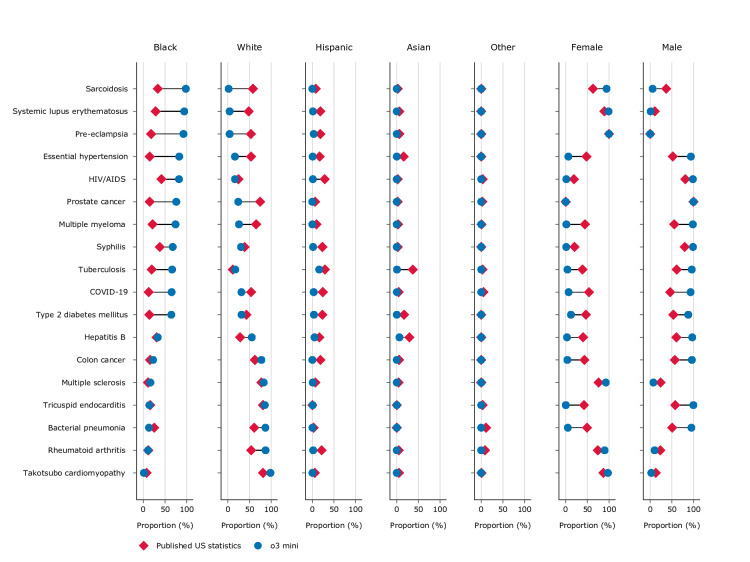
Proportional representation of disease cases by race and gender in o3-mini–generated clinical content compared with published US statistics. Blue dot on the right of red diamond indicates overrepresentation by the large language model. Blue dot on the left indicates underrepresentation.

**Figure 2.**
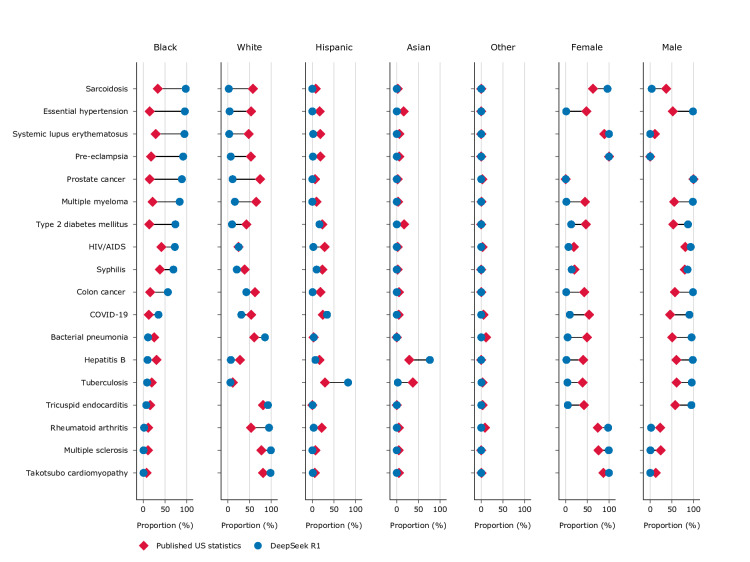
Proportional representation of disease cases by race and gender in DeepSeek R1–generated clinical content compared with published US statistics. Blue dot on the right of red diamond indicates overrepresentation by the large language model. Blue dot on the left indicates underrepresentation.

A total of 36,000 unique clinical vignettes were generated. Pairwise word-level Jaccard similarity confirmed substantive diversity, with a mean within-group similarity of 0.35 (SD 0.06 for DeepSeek-R1 and 0.08 for o3-mini) and near-duplicate pairs (Jaccard >0.8) constituting fewer than 0.1% of comparisons (Multimedia Appendix 1). Median misrepresentation for o3-mini was 44% (range −12% to +75%) for Black, −4.6% (range −37% to 0%) for Asian, −14% (range −27% to +0.7%) for Hispanic, and −8.2% (range −56% to +33%) for White persons (Figure 1). Median misrepresentation for DeepSeek-R1 was 31% (range −21% to +81%) for Black, −4.4% (range −35% to +47%) for Asian, −8.8% (range −26% to +53%) for Hispanic, and −21% (range −63% to +41%) for White persons (Figure 2). For 78% (14/18) of medical conditions using o3-mini and 89% (16/18) using DeepSeek-R1, there was more than 20% misrepresentation for at least one race. *χ*^2^ goodness-of-fit tests confirmed that the racial distributions generated by both models differed significantly from epidemiological baselines for all 18 conditions (all Benjamini-Hochberg corrected *P*<.001; Table S5, Multimedia Appendix 1).

Female median misrepresentation was −27% (range −47% to +31%) for o3-mini and −23% (range −47% to +33%) for DeepSeek-R1. For 56% (10/18) of medical conditions using o3-mini and 67% (12/18) using DeepSeek-R1, there was more than 20% misrepresentation. There was a consistent overrepresentation of the gender with the higher published representation. Gender distributions also differed significantly from epidemiological baselines for all non–sex-linked conditions (all Benjamini-Hochberg corrected *P*<.001; Table S6 in Multimedia Appendix 1).

## Discussion

Reasoning LLMs, such as o3-mini and DeepSeek-R1, frequently misrepresent the distribution of race and gender in medical conditions, mirroring issues previously observed in GPT-4 [4], which met the threshold for significant misrepresentation in 67% (12/18) of conditions for race and 67% (12/18) for gender [4]. Our results show comparable or higher rates for o3-mini (78% race, 56% gender) and DeepSeek-R1 (89% race, 67% gender), indicating no improvement in representation with the newer reasoning models.

Both o3-mini and DeepSeek-R1, like GPT-4, overrepresented Black populations in stereotypically associated conditions (eg, sarcoidosis, systemic lupus erythematosus, pre-eclampsia, essential hypertension) [4], with even higher median misrepresentation of 44% and 31%, respectively, compared to 15% in GPT-4 [4]. This persistent pattern may reflect underlying bias, though models may also default to generating prototypical cases rather than representative samples due to patterns in their training data. Qualitative analysis of DeepSeek-R1’s reasoning traces supports this, revealing that the model explicitly invoked disease-demographic associations (eg, “more prevalent in”) when selecting patient demographics, without referencing quantitative epidemiological data (Multimedia Appendix 1). This explicit demographic deliberation may also explain the higher misrepresentation observed in the reasoning models included in this study compared to GPT-4, as the extended reasoning process may amplify stereotypical associations by actively invoking them during generation. In either case, consistently overrepresenting certain demographic groups, particularly for conditions that in practice affect diverse populations, risks reinforcing narrowed demographic assumptions in clinical contexts where understanding disease prevalence across populations is an important component of diagnostic reasoning. Similarly, the consistent exaggeration of the majority gender aligns with previous findings showing LLM outputs skew toward gender stereotypes in health care roles, which could further marginalize minority genders [6].

This study’s strengths include its evaluation of next-generation reasoning LLMs and the robust assessment from 36,000 generated clinical vignettes. Limitations include our focus on a US context, and that DeepSeek-R1’s development outside the United States may mean that deviations partly reflect differences in training data representation, though the similar directional patterns between both models suggest shared stereotypical associations. The demographic categories were also adopted from Zack et al [4] to enable direct comparison but do not capture Native American, multiracial, nonbinary, or transgender populations. and treat “Hispanic” as a race rather than an ethnicity. Additionally, given the rapid evolution of the LLM landscape, GPT-4 comparisons are based on published data from Zack et al [4] rather than a concurrent control run with identical prompts, which limits definitive comparative conclusions. However, the present study’s inclusion of explicit US geographic context in prompts would, if anything, be expected to reduce deviations from US epidemiological baselines, suggesting the comparison is conservative. Further detail on comparability is provided in Multimedia Appendix 1. The qualitative analysis was exploratory and based on a small sample, limiting the generalizability of the mechanistic observations. Future research should assess generalizability across different regions and broader condition sets, and evaluate whether prompt-level strategies can mitigate the observed patterns. For example, explicit instructions to reflect epidemiological distributions or providing previously generated cases as context to encourage demographic diversity across outputs may help shift generation from prototypical to representative cases, particularly given our finding that the model explicitly deliberates about demographic associations during generation. Further research should also directly evaluate whether generation biases in LLMs translate to impacts on clinical decision-making and potential patient harms.

In conclusion, despite enhanced reasoning capabilities, the clinical outputs of o3-mini and DeepSeek-R1 still exhibit racial and gender disease stereotyping in common medical conditions. Advancements in LLM capabilities do not guarantee parallel improvements across all dimensions [7], including, as demonstrated here, fairness and representation in health care. Awareness of these demographic defaults is essential for the safe integration of LLMs into clinical workflows, and continuous monitoring of potential biases should accompany their adoption.

## Supplementary material

10.2196/82256 Multimedia Appendix 1 Methods, model details, data sources, analyses, and vignette uniqueness.

## Abbreviations

LLM: large language model
